# Hemophilia and hereditary angioedema: parallel therapeutic advances in genetic diseases of serine protease pathways

**DOI:** 10.1016/j.rpth.2026.103456

**Published:** 2026-03-26

**Authors:** Massimo Cugno, Pier Mannuccio Mannucci

**Affiliations:** 1 Università degli Studi di Milano, Department of Pathophysiology and Transplantation, Milan, SC Medicina-Emostasi e Trombosi, Fondazione IRCCS Ca’ Granda Ospedale Maggiore Policlinico, Milan, Italy; 2 Fondazione IRCCS Ca’ Granda Ospedale Maggiore Policlinico, Angelo Bianchi Bonomi Hemophilia and Thrombosis Center, Milan, Italy

**Keywords:** bradykinin, C1 inhibitor, emicizumab, factor VIII, factor IX, hemophilia, hereditary angioedema, kallikrein

## Abstract

Hemophilia and hereditary angioedema (HAE) are rare monogenic disorders characterized by the dysregulation of serine protease-based biological pathways, that is, blood coagulation and the kallikrein–kinin system. Although clinical manifestations differ profoundly (bleeding vs angioedema), both diseases have recently undergone parallel therapeutic revolutions, shaped by advances in molecular biology and biotechnology. Early management in the 1970s relied for both diseases on the episodic administration of plasma-derived products, subsequently replaced by recombinant products that improved safety and feasibility of prophylaxis regimens. In the last 20 years, the development of nonreplacement products, such as emicizumab and rebalancing agents in hemophilia and kallikrein/bradykinin pathway inhibitors in HAE, shifted clinical practice from the episodic management of clinical events to their prevention. More recently, gene and RNA-based therapies are further transforming both diseases toward curative attempts: in hemophilia, adeno-associated virus vector-mediated gene therapy and lentiviral stem-cell approaches; in HAE, antisense oligonucleotide–mediated kallikrein suppression. Emerging genome-editing approaches and biomarker- and genotype-driven strategies are poised to further improve and personalize treatment. The therapeutic trajectories of rare diseases such as hemophilia and HAE illustrate how mechanistic insights enable the transition from the episodic management of acute events to long-term disease control, offering prospects for curative interventions.

## Essentials

• Hemophilia and hereditary angioedema (HAE) are rare genetic diseases of serine protease pathways.
• The parallel evolution in treatments of both hemophilia and HAE is herewith presented comparing recent advances.
• Biologic drugs and rebalancing agents can now prevent bleeding in hemophilia and edema in HAE.
• Gene and RNA-based therapies may offer long-term control and future cures for both diseases.

## 1 Introduction

Hemophilia and hereditary angioedema (HAE), which are rare diseases of blood coagulation and kallikrein–kinin systems, exemplify how understanding molecular mechanisms leads to dramatic therapeutic advances. Hemophilia A and B are bleeding disorders caused by the plasma deficiency of coagulation factor (F)VIII (hemophilia A) or FIX (hemophilia B), stratified into severe, moderate, and mild forms on the basis of coagulation factor plasma levels. The deficiency or dysfunction of FVIII or FIX causes insufficient thrombin formation and a bleeding phenotype [1]. Bleeding episodes mainly recur in joints (hemarthrosis) and muscles (hematoma) and lead, if inadequately treated, to irreversible musculoskeletal damage and severe disability.

**Figure 1:**
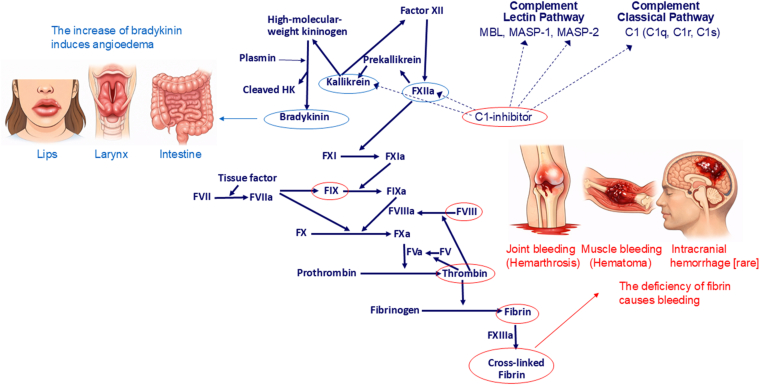
Simplified diagram of serine protease systems involved in hemophilia and hereditary angioedema due to C1-inhibitor deficiency. C1 inhibitor controls the activation of the classical and lectin pathways of the complement system by blocking C1r, C1s, MASP-1, and MASP-2. It is also the main inhibitor of the coagulation contact phase, which is initiated by the activation of factor (F)XII, occurring spontaneously or induced by negatively charged surfaces. Activated FXII (FXIIa) activates prekallikrein to kallikrein, which in turn activates FXII to FXIIa and cleaves high-molecular-weight kininogen (HK), releasing the vasoactive peptide bradykinin. The fibrinolytic enzyme plasmin enhances the release of bradykinin. When C1 inhibitor is deficient (circled in red), more FXIIa and kallikrein are produced (circled in blue), thus increasing bradykinin (circled in blue), that is, the ultimate cause of angioedema. The coagulation cascade—initiated by tissue factor/activated factor VII (FVIIa) and by the contact phase (kallikrein and FXIIa) followed by the sequential activation of the coagulation factors—leads to the formation of thrombin, which transforms fibrinogen into fibrin, stabilized by FXIII. When such critical factors of the coagulation system as FVIII or FIX are deficient (circled in red), there is an insufficient production of thrombin and ultimately of fibrin (circled in red), that is, the ultimate causes of bleeding.

Most cases of HAE are caused by the deficiency of C1 inhibitor (C1-INH), a large multifunctional protein that acts as an inhibitor of the complement component 1 (C1), as well as of kallikrein and activated FXII (FXIIa; Figure 1). C1-INH deficiency, which is inherited as an autosomal dominant trait, can be either quantitative (HAE type 1) or qualitative (HAE type 2) [2]. HAE has a prevalence of ∼1 in 50,000 in the general population [3] and is therefore rarer than hemophilia (∼1 in 10,000) [4]. C1-INH deficiency leads to enhanced conversion of prekallikrein to kallikrein, which cleaves high-molecular-weight kininogen to release the vasoactive peptide bradykinin (Figure 1) [5]. Bradykinin induces a reversible increase in vascular permeability, responsible for the formation of angioedema [6,7]. Angioedema manifests as a circumscribed, non-pitting edema of the subcutaneous tissues involving lips, face, neck, extremities, and/or the submucosal tissues of the oral cavity, larynx, and gastrointestinal tract. Laryngeal involvement may be life-threatening, and intestinal angioedema can be extremely painful and mimics an acute abdomen [8].

### 1.1 Disease mechanisms

Hemophilia A and B are due mainly in males to variants in the *F8* (hemophilia A) or *F9* (hemophilia B) genes on the X chromosome [1], whereas HAE is caused in both sexes by autosomal-dominant loss-of-function variants on chromosome 11 of the gene *SERPING1* (serine protease inhibitor family G member 1), which encodes C1-INH [2]. Rarely, forms of HAE unrelated to C1-INH deficiency have been described, owing to variants in other genes, including FXII (*F12*), plasminogen (*PLG*), angiopoietin-1 (*ANGPT1*), kininogen-1 (*KNG1*), myoferlin (*MYOF*), and heparan sulfate glucosamine 3-*O*-sulfotransferase-6 (*HS3ST6*) [9]. Although clinically different, bleeding vs angioedema, both diseases arise mechanistically from the dysregulation of tightly regulated serine protease systems (Figure 1). In them, a single disrupted protein causes abnormal downstream activity (more bradykinin in HAE and less thrombin in hemophilia), illustrating the critical importance of precise pathway regulation.

### 1.2 Clinical characteristics

Comparative clinical features of hemophilia and hereditary angioedema are shown in Table. Regarding the acute manifestations, they are usually not life-threatening in hemophilia, with the relatively rare exception of intracranial hemorrhages. In HAE, laryngeal edema and asphyxiation, historically dramatic problems, are no longer a life threat at least in high-income settings after the introduction of C1-INH replacement products. Long-term comorbidities are prominent in hemophilia, typically arthropathy and the consequences of hepatitis C infection, that is, cirrhosis and hepatocellular carcinoma [10]. Despite the historical importance for morbidity and mortality of hepatitis C virus and HIV infections in patients with hemophilia, blood screening techniques and the adoption of virucidal method for coagulation factor concentrates have made now extremely low the risk of acquiring new infections. In HAE, notwithstanding the occurrence of hepatitis C as a consequence of plasma-derived therapeutics, there is at the moment no solid evidence of late hepatic consequences [11]. Notably, a large population-based report recently found an increased risk of thrombosis (both arterial and venous) in people with genetically confirmed C1-INH deficiency [12]. These findings are biologically compatible with our earlier findings of hypercoagulability biomarkers in HAE [13]. There are also reports suggesting that C1-INH deficiency is associated with a higher risk of autoimmune diseases such as systemic lupus erythematosus and thyroiditis [14]. Overall, there are at least 2 reasons for an interest of experts of hemostasis and thrombosis on HAE, that is, the evidence of an increased risk of thrombosis and the fact that HAE is sometimes managed in specialized hemophilia centers.

**Table:** Comparative features of hemophilia and hereditary angioedema.

Feature	Hemophilia	Hereditary angioedema
Serine protease system	Coagulation	Kallikrein–kinin
Protein deficiency or dysfunction	Factor (F)VIII or FIX	C1 inhibitor
Genetics	Mutations in *F8* (hemophilia A) or *F9* (hemophilia B) genes on the X chromosome	Mutations mainly in *SERPING1* gene encoding C1 inhibitor
Inheritance pattern	X-linked recessive	Autosomal dominant
Sex distribution	Predominantly in males; female carriers may be symptomatic	Both sexes equally affected
Disease mechanism	Thrombin deficiency → defective fibrin clot	Uncontrolled kallikrein → bradykinin excess
Disease classification	Severe (<1% factor activity), moderate (1%-5%), mild (>5%-40%) based on circulating FVIII/FIX levels	Type 1: C1-inhibitor deficiency Type 2: C1-inhibitor dysfunction No universally accepted biochemical severity classification
Acute clinical manifestations	Bleeding (hemarthroses, muscle hematomas, and posttraumatic or spontaneous bleeding)	Angioedema (episodic swelling of subcutaneous or submucosal tissues: skin, upper airways, and gastrointestinal tract)
Reversibility of damage	Repeated hemarthroses lead to irreversible arthropathy and disability	Complete resolution of attacks; swelling reversible with no permanent tissue damage
Mortality risk	Much reduced with modern therapies; historically due to intracranial or uncontrolled bleeding and transfusion-transmitted infections	Historically due to laryngeal edema and asphyxiation; dramatically reduced by currently available therapies
Complications	Chronic arthropathy, musculoskeletal disability, inhibitor development	Impaired quality of life, anxiety related to unpredictable attacks; no structural organ damage. Risk of arterial and venous thrombosis
Inhibitor development	Frequent in hemophilia A (∼25% to 35% in severe cases)	Antibodies to C1 inhibitor extremely rare after replacement
Risk of infections	Historically high HBV/HCV/HIV transmission from plasma-derived concentrates	Low; limited number of cases
Liver sequelae	Chronic viral hepatitis, cirrhosis, and hepatocellular carcinoma in older cohorts	Uncommon

HBV, hepatitis B virus; HCV, hepatitis C virus.

### 1.3 Therapeutics

**Figure 2:**
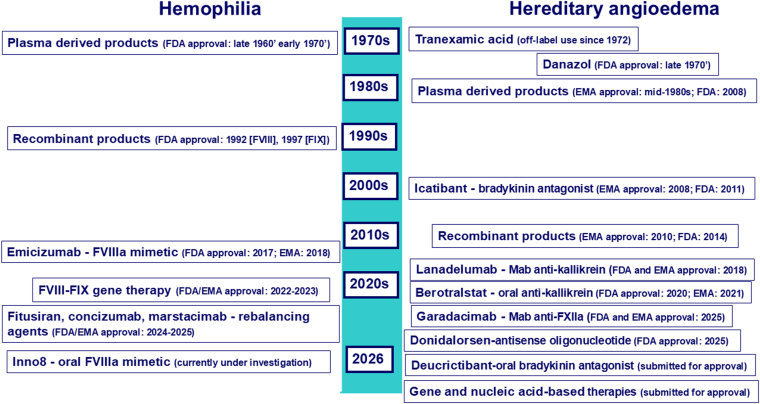
Evolution of therapies for hemophilia and hereditary angioedema. Both diseases evolved from plasma-derived replacement therapies to recombinant and targeted biological treatments, culminating in gene-based strategies. The years of approval of the therapies are shown in parentheses for both the European Medicines Agency (EMA) and the Food and Drug Administration (FDA).

Throughout the past 2 decades, therapeutic innovations have been profound for both diseases, with a related dramatic improvement in life expectancy, now similar to that of unaffected peers [10,15]. Initial treatment of the acute manifestations of bleeding in hemophilia and attacks of edema in HAE evolved for both diseases from the episodic intravenous administration of plasma-derived products to those manufactured by recombinant DNA technology and, more recently, to long-acting recombinant molecules, subcutaneous biologics, oral agents, RNA-based drugs, and gene therapy (Figure 2). Thus, hemophilia and HAE stand as parallel examples of rare diseases leading to a broader strategy of precision medicine, with the goal to prevent disease manifestations and thus improve both life expectancy and quality of life. With this background, the goal of this review article is to compare the parallel evolution that occurred mainly in the last 2 decades for both diseases and to emphasize shared therapeutic progress (notwithstanding divergent molecular targets).

## 2 Search Methodology

The literature was identified using PubMed and Google Scholar searches for articles published between 1960 and 2025 using the terms hemophilia, hereditary angioedema, treatment, gene therapy, and precision medicine. Only high-quality articles have been selected, and emphasis was placed on clinical trials, major review articles, and mechanistic studies. Information on emerging therapies was supplemented with data from ClinicalTrials.gov. The structure of the review reflects conceptual themes rather than strict chronology.

## 3 Therapeutic Evolution: From Replacement to Modulation

Figure 2 depicts the timeline of available therapies for both hemophilia and HAE and the progress from plasma-derived and recombinant replacement products to nonreplacement drugs toward gene and nucleic acid-based therapeutics. As highlighted, in some settings, the specialized management of both diseases takes place in hemophilia treatment centers, owing to their proficiency in replacement therapy and expertise accrued in the 1970s and 1980s regarding the education of patients and families to self-treatment and handle injections.

### 3.1 Plasma-derived and recombinant replacement therapies

In hemophilia, early treatment (1964-1990s) was based on plasma-derived factor replacement on the occasion of bleeding episodes, first with cryoprecipitate and then with large plasma pool concentrates of the deficient FVIII and FIX. These therapeutics were a major breakthrough in the 1970s, subsequently offset in the 1980s by the transmission of such viral bloodborne disease as hepatitis and AIDS. A pharmacologic therapy such as desmopressin was developed in the late 1970s, but its use is restricted to patients with mild hemophilia A but not to those with hemophilia B.

Unsatisfactory clinical efficacy, with the related almost inevitable development of musculoskeletal complications and invalidating handicaps, drove innovation from episodic treatment toward prophylaxis of bleeding [16]. The application of virucidal methods to plasma-derived products and then the introduction of recombinant FVIII and FIX in the 1990s greatly improved safety and availability, at least in high-income countries [17,18]. Extended plasma half-life recombinant coagulation factors (engineered using PEGylation, Fc-fusion, or albumin-fusion technologies) were developed in the 2000s [1] and enabled prophylaxis with less frequent intravenous infusions, that is, every 1 to 2 weeks for FIX and every 3 to 5 days for FVIII.

A similar transition occurred in HAE. Plasma-derived C1-INH concentrates such as Berinert and Cinryze have been available since the 1980s, enabling episodic therapy of angioedema attacks [19] and their prevention [20]. The subsequent availability of recombinant C1-INH (Ruconest), purified from the milk of transgenic rabbits, was triggered by the transmission with plasma-derived products of bloodborne viral infections and offered also in HAE an alternative to plasma [20]. Thus, both diseases moved from blood products to recombinant DNA technology, leading to improved safety, efficacy, and availability, at least in high-income countries. In addition, both helped to pioneer the regulatory and manufacturing scenario of replacement therapies of rare plasma proteins.

### 3.2 Nonreplacement therapies

Historically, the first nonreplacement pharmacologic therapies for patients with mild hemophilia A were intravenous, subcutaneous, or intranasal desmopressin (1-deamino-8-d-arginine vasopressin) [21] and, for HAE, the oral antifibrinolytic drug tranexamic acid [22] and the attenuated androgens stanozolol and danazol [23]. In hemophilia A, the most striking advance that materialized in the last 10 years was emicizumab, a humanized bispecific antibody that mimics the coagulant activity of activated FVIII by bridging FIXa and FX [24]. It allows subcutaneous dosing every 1 to 4 weeks, is efficacious in both FVIII inhibitor-positive and inhibitor-negative patients, and dramatically transformed the paradigm and feasibility of prophylaxis [1]. Additionally, more recent approaches are rebalancing agents, tailored to enhance the production of thrombin by quenching the activity of naturally occurring anticoagulant proteins. This can be obtained by silencing the gene of antithrombin with fitusiran or blocking tissue factor pathway inhibitor, with monoclonal antibodies such as concizumab and marstacimab, all aimed at restoring defective thrombin formation without the need of coagulation factor replacement [1]. These subcutaneous drugs are effective in both the hemophilias, with and without coagulation factor inhibitors.

In HAE, understanding that bradykinin is the ultimate trigger of edema attacks [6,7] led to therapeutics targeting specifically the kallikrein–bradykinin pathway. Icatibant, a B2 bradykinin receptor antagonist, provides on-demand treatment for acute episodes [25]. Inhibition of kallikrein activity can be obtained by ecallantide, a recombinant protein synthesized in the yeast *Pichia pastoris*, which administered subcutaneously improves acute edema symptoms [26]. Lanadelumab, a subcutaneously administered, human monoclonal antibody against plasma kallikrein, is used for prophylaxis of swelling attacks and demonstrated long-term efficacy [27]. Moreover, the monoclonal antibody directed against FXIIa garadacimab, when administered subcutaneously once a month, reduces the frequency of HAE attacks with a good safety profile [28]. Berotralstat (BCX7353), the first oral kallikrein inhibitor, offers daily prophylaxis with good tolerability [29]. Deucrictibant, an orally bioavailable bradykinin B2 receptor antagonist, is currently being evaluated for prophylaxis in phase 2 to 3 studies, as an extension of studies for the acute treatment of angioedema attacks (NCT05396105). In hemophilia, oral therapies are lagging behind those for HAE, but Inno8 is a small FVIIIa mimetic antibody, engineered to make it orally adsorbable and circulation available. It is currently in phase 1 clinical trial to prove safety in healthy males (NCT06649630). All these therapeutics are efficacious by acting on the ultimate downstream mediators of clinical manifestations, that is inhibiting kallikrein and bradykinin in HAE and enhancing thrombin formation in hemophilia.

### 4 Gene and Nucleic Acid-Based Therapies

In hemophilia, the advent of gene therapy is shifting management toward cure. Adeno-associated virus vector-mediated delivery of FVIII (valoctocogene roxaparvovec) or FIX transgenes (etranacogene dezaparvovec) achieves sustained endogenous coagulation factor production, thus reducing or eliminating in recipients the need of prophylaxis with replacement or nonreplacement products and ultimately pursuing cure [30,31]. Although concerns remain particularly in hemophilia A regarding variable transgene expression, FVIII plasma level durability, immune-mediated responses and related hepatotoxicity, results from clinical trials demonstrate substantial benefits. Lentiviral hematopoietic stem cell gene therapy has been recently pioneered in hemophilia A as an alternative vector platform with potentially greater genomic stability [32].

In HAE, gene therapy is less advanced than in hemophilia, but nucleic acid-based therapies are rapidly progressing. Donidalorsen, an antisense oligonucleotide targeting prekallikrein mRNA, achieved durable reductions of angioedema attacks and was approved by regulatory agencies [33,34]. This therapy aligns conceptually with approaches that, in hemophilia, use RNA silencing to quench the activity of naturally occurring anticoagulant proteins. Fitusiran is small-interfering RNA that specifically targets antithrombin mRNA to suppress the production of antithrombin in the liver. Its monthly or bimonthly subcutaneous administration lowered annual bleed rates in patients with hemophilia A or B with and without inhibitors [1]. Together, these developments point toward longer-acting therapies. While in hemophilia, gene therapy restores the endogenous production of the deficient proteins, in HAE, nucleic acid-based therapies typically aim to suppress overactive enzymatic pathways.

## 5 Emerging Frontiers and Future Directions

Two major themes dominate the next wave of innovation: gene editing and individualized precision medicine.

### 5.1 Gene editing approaches

Gene editing technologies such as CRISPR/Cas9 (clustered regularly interspaced short palindromic repeats and CRISPR-associated protein 9), base editing, and prime editing are transforming the landscape of genetic medicine. In hemophilia, preclinical studies demonstrated the successful correction of F8 and F9 variants in hepatocytes, as well as gene editing of hematopoietic stem cells. In hemophilia B, an open-label study is ongoing for REGV131-LNP1265, a CRISPR/Cas9-based F9 gene insertion therapy (NCT06379789). These strategies aim for a 1-time, permanent correction of the genetic defect, overcoming the limitations of adeno-associated virus vector-mediated approaches, which rely on episomal transgene expression that tends to vanish over time. Another innovative approach involves platelet-targeted gene editing, based upon megakaryocytes, tailored to express FVIII in platelets that release it locally at sites of vascular injury (NCT03818763). Encouraging results were reported in the first enrolled case, that is, a 29-year-old man with hemophilia A and a history of FVIII inhibitor [35].

In HAE, an ongoing study (NCT05120830) is evaluating NTLA-2002, a nonviral gene-editing therapy based on CRISPR/Cas9, which targets the gene encoding kallikrein. The results of the phase 2 portion of the study show that NTLA-2002, administered as a single dose, reduced angioedema attacks through the reduction of plasma kallikrein levels [36].

Therefore, both disorders—hemophilia and HAE—moved from acute symptom management to genetic repair. Hemophilia is more advanced along this pathway, but HAE is entering the same conceptual territory, demonstrating how rare diseases lead to innovation across molecular therapies.

### 5.2 Precision medicine

Precision medicine has been defined as treatments targeted to the needs of individual patients on the basis of genetic, phenotypic, or psychosocial characteristics that distinguish a patient from others with similar clinical presentations [37]. In hemophilia, the genotype strongly predicts inhibitor incidence, the main still unresolved clinical complication. The highest incidence is seen in patients with null variants such as large gene deletions and intron 22 inversion, whereas missense variants have the lowest incidence [38]. Personalized prophylaxis can be guided by the chosen hemostatic treatment regimen, quality of life, presence or absence of joint disease, and pharmacokinetic studies [1].

In HAE, evidence that accumulated over the last few decades indicates that genotype–phenotype correlations may influence therapeutic decisions [39]. Several biochemical and genetic biomarkers offer decision-making value on the basis of their long-term empirical use for the episodic treatment of edema attacks and their long-term prevention [34,39]. Emerging biomarkers, including the measurement of plasma kallikrein activity and bradykinin, may further tailor therapeutic choices and dosages. Precision medicine in both hemophilia and HAE can also leverage digital tools, event diaries, and stress monitoring to predict the risk of bleeds or angioedema attacks, thus enabling more personalized treatment.

## 6 Conclusion

Hemophilia and HAE exemplify parallel therapeutic revolutions driven by advances in molecular biology and biotechnology. Both disorders have progressed from plasma-derived therapeutics to recombinant biologics, targeted monoclonal antibodies, oral products, and more recently gene therapy. In addition, emerging gene-editing technologies offer the prospect of 1-time curative interventions. Despite their largely different clinical manifestations, disease complications, and comorbidities, the therapeutic trajectories of hemophilia and HAE demonstrate how mechanistic insights can reshape management. In both diseases, therapeutic evolutions have truly informed each other: hemophilia informed the use of replacement therapy in HAE, while agents acting downstream on kallikrein–bradykinin in HAE informed the advent of thrombin rebalancing agents in hemophilia.

There are specialized clinical centers that manage both diseases. This approach is historically based upon the fact that hemophilia specialists had accrued long-term expertise pertaining to patient education to self-treatment and training to carry out injections. Moreover, the interest of hemostasis and thrombosis experts regarding the mechanistic role of C1-INH and its deficiency is enhanced by the more and more clear role of C1-INH deficiency as a risk factor for venous and arterial thrombosis.

## References

[1] Chowdary P., Carcao M., Kenet G., Pipe S.W. (2025). Haemophilia. Lancet.

[2] Reshef A., Buttgereit T., Betschel S.D., Caballero T., Farkas H., Grumach A.S. (2024). Definition, acronyms, nomenclature, and classification of angioedema (DANCE): AAAAI, ACAAI, ACARE, and APAAACI DANCE consensus. J Allergy Clin Immunol.

[3] Castaldo A.J., Wells K.E., Khalid S., Rashidi E., Selva C.N., Corcoran D. (2025). Establishing a hereditary angioedema prevalence for the United States using a large administrative claims database. Ann Allergy Asthma Immunol.

[4] Iorio A., Stonebraker J.S., Chambost H., Makris M., Coffin D., Herr C. (2019). Establishing the prevalence and prevalence at birth of hemophilia in males: a meta-analytic approach using national registries. Ann Intern Med.

[5] Cugno M., Zanichelli A., Foieni F., Caccia S., Cicardi M. (2009). C1-inhibitor deficiency and angioedema: molecular mechanisms and clinical progress. Trends Mol Med.

[6] Nussberger J., Cugno M., Amstutz C., Cicardi M., Pellacani A., Agostoni A. (1998). Plasma bradykinin in angio-oedema. Lancet.

[7] Nussberger J., Cugno M., Cicardi M. (2002). Bradykinin-mediated angioedema. N Engl J Med.

[8] Depetri F., Tedeschi A., Cugno M. (2019). Angioedema and emergency medicine: from pathophysiology to diagnosis and treatment. Eur J Intern Med.

[9] Zuraw B.L., Bork K., Bouillet L., Christiansen S.C., Farkas H., Germenis A.E. (2025). Hereditary angioedema with normal C1 inhibitor: an updated international consensus paper on diagnosis, pathophysiology, and treatment. Clin Rev Allergy Immunol.

[10] Rühl H., Marquardt N., Oldenburg J. (2026). Ageing with haemophilia: comorbidities in focus. Transfus Med Hemother.

[11] Zanichelli A., Senter R., Merlo A., Gidaro A., Popescu Janu V., Cogliati C.B. (2024). Comorbidities in angioedema due to C1-inhibitor deficiency: an Italian survey. J Allergy Clin Immunol Pract.

[12] Rodriguez Espada A., Haj A.K., Jurgens S.J., Eswaran H., Sundler Björkman L., Ryu J. (2026). Population-scale analysis reveals inherited C1-inhibitor deficiency is a polyphenotypic thrombotic disorder. Blood Adv.

[13] Cugno M., Cicardi M., Bottasso B., Coppola R., Paonessa R., Mannucci P.M. (1997). Activation of the coagulation cascade in C1-inhibitor deficiencies. Blood.

[14] Levy D., Craig T., Keith P.K., Krishnarajah G., Beckerman R., Prusty S. (2020). Co-occurrence between C1 esterase inhibitor deficiency and autoimmune disease: a systematic literature review. Allergy Asthma Clin Immunol.

[15] Perego F., Gidaro A., Zanichelli A., Cancian M., Arcoleo F., Senter R. (2020). Life expectancy in Italian patients with hereditary angioedema due to C1-inhibitor deficiency. J Allergy Clin Immunol Pract.

[16] Pierce GF, Lusher JM, Brownstein AP, Goldsmith JC, Kessler CM. The use of purified clotting factor concentrates in hemophilia. Influence of viral safety, cost, and supply on therapy. JAMA 261:3434–3438. 2498537

[17] Schwartz RS, Abildgaard CF, Aledort LM, Arkin S, Bloom AL, Brackmann HH, et al. Human recombinant DNA-derived antihemophilic factor (factor VIII) in the treatment of hemophilia A. Recombinant Factor VIII Study Group. N Engl J Med 323:1800–1805. 10.1056/NEJM199012273232604 2123300

[18] White G.C., Beebe A., Nielsen B. (1997). Recombinant factor IX. Thromb Haemost.

[19] Gadek J.E., Hosea S.W., Gelfand J.A., Santaella M., Wickerhauser M., Triantaphyllopoulos D.C. (1980). Replacement therapy in hereditary angioedema: successful treatment of acute episodes of angioedema with partly purified C1 inhibitor. N Engl J Med.

[20] Betschel S.D., Banerji A., Busse P.J., Cohn D.M., Magerl M. (2023). Hereditary angioedema: a review of the current and evolving treatment landscape. J Allergy Clin Immunol Pract.

[21] Mannucci P.M., Ruggeri Z.M., Pareti F.I., Capitanio A. (1977). 1-Deamino-8-d-arginine vasopressin: a new pharmacological approach to the management of haemophilia and von Willebrands’ diseases. Lancet.

[22] Sheffer A.L., Austen K.F., Rosen F.S. (1972). Tranexamic acid therapy in hereditary angioneurotic edema. N Engl J Med.

[23] Gelfand J.A., Sherins R.J., Alling D.W., Frank M.M. (1976). Treatment of hereditary angioedema with danazol. Reversal of clinical and biochemical abnormalities. N Engl J Med.

[24] Kitazawa T., Igawa T., Sampei Z., Muto A., Kojima T., Soeda T. (2012). A bispecific antibody to factors IXa and X restores factor VIII hemostatic activity in a hemophilia A model. Nat Med.

[25] Cicardi M., Banerji A., Bracho F., Malbrán A., Rosenkranz B., Riedl M. (2010). Icatibant, a new bradykinin-receptor antagonist, in hereditary angioedema. N Engl J Med.

[26] Cicardi M., Levy R.J., McNeil D.L., Li H.H., Sheffer A.L., Campion M. (2010). Ecallantide for the treatment of acute attacks in hereditary angioedema. N Engl J Med.

[27] Banerji A., Bernstein J.A., Johnston D.T., Lumry W.R., Magerl M., Maurer M. (2022). Long-term prevention of hereditary angioedema attacks with lanadelumab: the HELP OLE study. Allergy.

[28] Craig T.J., Reshef A., Li H.H., Jacobs J.S., Bernstein J.A., Farkas H. (2023). Efficacy and safety of garadacimab, a factor XIIa inhibitor for hereditary angioedema prevention (VANGUARD): a global, multicentre, randomised, double-blind, placebo-controlled, phase 3 trial. Lancet.

[29] Aygören-Pürsün E., Bygum A., Grivcheva-Panovska V., Magerl M., Graff J., Steiner U.C. (2018). Oral plasma kallikrein inhibitor for prophylaxis in hereditary angioedema. N Engl J Med.

[30] Pasi K.J., Rangarajan S., Mitchell N., Lester W., Symington E., Madan B. (2020). Multiyear follow-up of AAV5-hFVIII-SQ gene therapy for hemophilia A. N Engl J Med.

[31] Reiss U.M., Davidoff A.M., Tuddenham E.G.D., Chowdary P., McIntosh J., Muczynski V. (2025). Sustained clinical benefit of AAV gene therapy in severe hemophilia B. N Engl J Med.

[32] Srivastava A., Abraham A., Aboobacker F., Singh G., Geevar T., Kulkarni U. (2025). Lentiviral gene therapy with CD34+ hematopoietic cells for hemophilia A. N Engl J Med.

[33] Riedl M.A., Tachdjian R., Lumry W.R., Craig T., Karakaya G., Gelincik A. (2024). Efficacy and safety of donidalorsen for hereditary angioedema. N Engl J Med.

[34] Ameratunga R., Longhurst H.J. (2024). New therapies for type 1 and type 2 hereditary angioedema. N Engl J Med.

[35] Eapen M., Malec L.M., Armant M.A., Johnson B.D., Shi Q., Xu H. (2025). Platelet-targeted gene therapy for hemophilia A with inhibitor history. N Engl J Med.

[36] Cohn D.M., Gurugama P., Magerl M., Katelaris C.H., Launay D., Bouillet L. (2025). CRISPR-based therapy for hereditary angioedema. N Engl J Med.

[37] Jameson J.L., Longo D.L. (2015). Precision medicine—personalized, problematic, and promising. N Engl J Med.

[38] Andersson N.G., Labarque V., Kartal-Kaess M., Pinto F., Mikkelsen T.S., Ljung R. (2024). Factor VIII genotype and the risk of developing high-responding or low-responding inhibitors in severe hemophilia A: data from the PedNet Hemophilia Cohort of 1,202 children. Haematologica.

[39] Germenis A.E., Cicardi M. (2019). Driving towards precision medicine for angioedema without wheals. J Autoimmun.

